# Supplementary material for: Potent Antiviral Activity of Vitamin B12 against Severe Acute Respiratory Syndrome Coronavirus 2, Middle East Respiratory Syndrome Coronavirus, and Human Coronavirus 229E
Source: Microorganisms. 2023 Nov 15;11(11):2777. doi: 10.3390/microorganisms11112777 (PMC10673013; doi:10.3390/microorganisms11112777)
Supplement: Supplementary file 1 [file microorganisms-11-02777-s001.zip › microorganisms-2633835-supplementary.pdf]

# Potent Antiviral Activity of Vitamin B12 against Severe Acute Respiratory Syndrome Coronavirus 2, Middle East Respiratory Syndrome Coronavirus, and Human Coronavirus 229E

## Supplementary Data

Table S1: Binding energies (as Docking and Glide scores) of the tested vitamins against RBD and spike proteins correlated with the three coronaviruses. All score values are in negative charge, as performed by Glide software (Schrödinger Release 2020-3: Glide; Schrödinger, LLC, New York, NY, USA, 2020). Modeled spike proteins of SARS-Cov-2 and MERS-CoV are based on the sequence of Egyptian strains used for In-Vitro testing in this study. The CoV-19/Egypt/NRC-03/2020 SARS-CoV-2 (GISAID number: EPI_ISL_430819), NRCE-HKU270 MERS-CoV (Genbank Accession: KJ477103.2) . while the others are 3D structures imported from protein database PDB).

| | | SARS CoV 2 _ S pro | | SARS CoV 2 _ S pro modeled | | MERS _ RBD | | MERS _ RBD _ modeled | | 229E _ S pro | |
|---|---|---|---|---|---|---|---|---|---|---|---|
| **Chemical name** | **vitamin** | **docking score (kcal/mol)** | **Glide score (kcal/mol)** | **docking score (kcal/mol)** | **Glide score (kcal/mol)** | **docking score (kcal/mol)** | **Glide score (kcal/mol)** | **docking score (kcal/mol)** | **Glide score (kcal/mol)** | **docking score (kcal/mol)** | **Glide score (kcal/mol)** |
| **Alfacalcidol** | **D3** | -4.7 | -4.7 | -5.8 | -5.8 | -3.0 | -3.0 | -3.2 | -3.2 | -4.2 | -4.2 |
| **Alpha Lipoic Acid** | | -3.8 | -3.8 | -4.4 | -4.4 | -2.7 | -2.7 | -2.7 | -4.4 | -3.0 | -3.0 |
| **Ascorbic Acid** | **C** | -4.9 | -4.9 | -4.9 | -4.9 | -3.2 | -3.2 | -3.9 | -3.9 | -3.8 | -3.8 |
| **Benfotiamine** | **B1** | -4.2 | -4.3 | -5.9 | -6.0 | -3.2 | -3.4 | -3.5 | -3.7 | -4.8 | -6.4 |
| **Biotin** | **B7** | -5.4 | -5.4 | -5.2 | -5.2 | -3.6 | -3.6 | -3.6 | -3.6 | -4.5 | -4.5 |
| **Cholecalciferol** | **D3** | -3.9 | -3.9 | -5.1 | -5.1 | -3.0 | -3.0 | -3.0 | -3.0 | -4.0 | -4.0 |
| **Cyanocobalamin** | **B12** | -9.5 | -9.5 | No interaction | No interaction | -7.3 | -7.4 | -4.3 | -5.6 | -10.6 | -10.7 |
| **Ergocalciferol** | **D2** | -4.3 | -4.3 | -4.8 | -4.8 | -3.5 | -3.5 | -3.0 | -3.0 | -3.8 | -3.8 |
| **Folic Acid** | **B9** | -6.0 | -6.2 | -5.8 | -6.1 | -4.7 | -5.0 | -5.6 | -5.9 | -6.4 | -6.6 |
| **Hydroxocobalamin** | **B12** | -9.5 | -9.5 | No interaction | No interaction | -7.3 | -7.4 | -4.3 | -5.6 | -10.6 | -10.7 |
| **Methylcobalamin** | **B12** | -9.2 | -9.3 | No interaction | No interaction | -7.2 | -7.3 | -5.4 | -5.5 | -9.2 | -9.3 |
| **Niacinamide** | **B3** | -6.0 | -6.0 | -5.9 | -5.9 | -4.3 | -4.3 | -4.3 | -4.3 | -5.1 | -5.1 |
| **Pantothenic Acid** | **B5** | -1.4 | -1.4 | -1.9 | -1.9 | -0.6 | -0.6 | -1.1 | -1.1 | -1.7 | -1.7 |
| **Phytomenadione** | **K1** | -3.8 | -3.8 | -4.4 | -4.4 | -1.6 | -1.6 | -2.0 | -2.0 | -3.3 | -3.3 |
| **Pyridoxal** | **B6** | -5.0 | -5.0 | -3.6 | -3.6 | -3.7 | -3.7 | -4.3 | -4.4 | -5.5 | -5.5 |
| **Retinol** | **A** | -3.0 | -3.0 | -4.5 | -4.5 | -3.0 | -3.0 | -2.9 | -2.9 | -2.5 | -2.5 |
| **Riboflavin** | **B2** | -6.0 | -6.0 | -6.0 | -6.0 | -3.5 | -3.5 | -4.1 | -4.1 | -5.7 | -5.7 |
| **Thiamine** | **B1** | -4.6 | -4.6 | -5.2 | -5.2 | -4.1 | -4.1 | -3.6 | -3.7 | -5.5 | -5.5 |
| **Tocopherols** | **E** | -3.7 | -3.7 | -4.6 | -4.6 | -2.6 | -2.6 | -2.9 | -2.9 | -4.1 | -4.1 |
| **Tocopheryl Acetate** | | -3.2 | -3.2 | -3.5 | -3.5 | -2.0 | -2.0 | -2.1 | -2.1 | -3.4 | -3.4 |
| **Vitamin K3** | **K3** | -6.1 | -6.1 | -5.3 | -5.3 | -3.7 | -3.7 | No interaction | No interaction | -4.4 | -4.4 |

Table S2: Binding energies (as Docking and Glide scores) of the tested vitamins against 3C-like protease (3CLpro) or main protease correlated with the three coronaviruses. All score values are in negative charge, as performed by Glide software (Schrödinger Release 2020-3: Glide; Schrödinger, LLC, New York, NY, USA, 2020). Modeled proteins of MERS-CoV are based on the sequence of Egyptian strains used for In-Vitro testing in this study, NRCE-HKU270 MERS-CoV (Genbank Accession: KJ477103.2) . while the others are 3D structures imported from protein database PDB).

| | | SARS CoV 2 _ 3CL | | MERS _ 3CL | | MERS _ 3CL _ modeled | | 229E _ 3CL | |
|---|---|---|---|---|---|---|---|---|---|
| **chemical name** | **vitamin** | **docking score (kcal/mol)** | **Glide score (kcal/mol)** | **docking score (kcal/mol)** | **Glide score (kcal/mol)** | **docking score (kcal/mol)** | **Glide score (kcal/mol)** | **docking score (kcal/mol)** | **Glide score (kcal/mol)** |
| **Alfacalcidol** | **D3** | -4.896 | -4.896 | -6.833 | -6.833 | -5.951 | -5.951 | -5.995 | -5.995 |
| **Alpha Lipoic Acid** | | -3.664 | -3.667 | -5.179 | -5.182 | -4.862 | -4.865 | -4.651 | -4.654 |
| **Ascorbic Acid** | **C** | -4.399 | -4.399 | -4.808 | -4.808 | -4.89 | -4.89 | -4.293 | -4.293 |
| **Benfotiamine** | **B1** | -3.796 | -3.931 | -7.504 | -7.639 | -5.235 | -5.37 | -3.828 | -5.483 |
| **Biotin** | **B7** | -4.757 | -4.76 | -6.265 | -6.268 | -7.191 | -7.194 | -4.203 | -4.206 |
| **Cholecalciferol** | **D3** | -4.293 | -4.293 | -6.215 | -6.215 | -5.636 | -5.636 | -5.698 | -5.698 |
| **Cyanocobalamin** | **B12** | No interaction | No interaction | -10.185 | -10.252 | -10.2 | -10.267 | No interaction | No interaction |
| **Ergocalciferol** | **D2** | -4.501 | -4.501 | -6.451 | -6.451 | -5.781 | -5.781 | -6.001 | -6.001 |
| **Folic Acid** | **B9** | -6.177 | -6.456 | -7.868 | -8.148 | -6.992 | -7.272 | -4.008 | -4.287 |
| **Hydroxocobalamin** | **B12** | No interaction | No interaction | -10.181 | -10.248 | -10.2 | -10.267 | No interaction | No interaction |
| **Methylcobalamin** | **B12** | No interaction | No interaction | -10.634 | -10.704 | -7.848 | -9.172 | No interaction | No interaction |
| **Niacinamide** | **B3** | -4.844 | -4.844 | -5.796 | -5.796 | -5.492 | -5.492 | -4.985 | -4.985 |
| **Pantothenic Acid** | **B5** | -2.046 | -2.047 | -3.218 | -3.22 | -2.764 | -2.765 | -1.825 | -1.826 |
| **Phytomenadione** | **K1** | -3.553 | -3.553 | -5.776 | -5.776 | -4.294 | -4.294 | No interaction | No interaction |
| **Pyridoxal** | **B6** | -4.172 | -4.21 | -5.833 | -5.872 | -6.181 | -6.22 | -4.952 | -4.991 |
| **Retinol** | **A** | -3.391 | -3.391 | -4.933 | -4.933 | No interaction | No interaction | -4.542 | -4.542 |
| **Riboflavin** | **B2** | -4.763 | -4.763 | -6.802 | -6.803 | -7.924 | -7.924 | -5.829 | -5.829 |
| **Thiamine** | **B1** | -5.279 | -5.298 | -6.926 | -6.945 | -6.244 | -6.263 | -5.279 | -5.298 |
| **Tocopherols** | **E** | -3.552 | -3.552 | -5.763 | -5.763 | -4.793 | -4.793 | -4.744 | -4.744 |
| **Tocopheryl Acetate** | | -3.574 | -3.574 | -5.168 | -5.168 | -4.595 | -4.595 | -4.51 | -4.51 |
| **Vitamin K3** | **K3** | -4.776 | -4.776 | -6.628 | -6.628 | -6.277 | -6.277 | No interaction | No interaction |

Table S3: Binding energies (as Docking and Glide scores) of the tested vitamins against RNA dependent RNA polymerase (RdRp)correlated with the three coronaviruses. All score values are in negative charge, as performed by Glide software (Schrödinger Release 2020-3: Glide; Schrödinger, LLC, New York, NY, USA, 2020). Modeled proteins of SARS-Cov-2 and MERS-CoV are based on the sequence of Egyptian strains used for In-Vitro testing in this study. The CoV-19/Egypt/NRC-03/2020 SARS-CoV-2 (GISAID number: EPI_ISL_430819), NRCE-HKU270 MERS-CoV (Genbank Accession: KJ477103.2) . while the others are 3D structures imported from protein database PDB).

| | | **SARS CoV 2 _ RdRp** | | **SARS CoV 2_RdRp_modeled** | | **MERS _ RdRp_modeled** | | **229E_RdRp_modeled** | |
|---|---|---|---|---|---|---|---|---|---|
| **chemical name** | **vitamin** | **docking score (kcal/mol)** | **Glide score (kcal/mol)** | **docking score (kcal/mol)** | **Glide score (kcal/mol)** | **docking score (kcal/mol)** | **Glide score (kcal/mol)** | **docking score (kcal/mol)** | **Glide score (kcal/mol)** |
| **Alfacalcidol** | **D3** | -4.54 | -4.54 | -4.104 | -4.104 | -4.159 | -4.159 | -4.974 | -4.974 |
| **Alpha Lipoic Acid** | | -3.656 | -3.659 | -4.242 | -4.242 | -3.747 | -3.75 | -3.322 | -3.352 |
| **Ascorbic Acid** | **C** | -3.534 | -3.534 | -4.51 | -4.51 | -5.117 | -5.117 | -4.856 | -4.856 |
| **Benfotiamine** | **B1** | -5.397 | -5.532 | -4.6 | -4.735 | -4.971 | -5.106 | -3.785 | -3.92 |
| **Biotin** | **B7** | -3.337 | -3.337 | -4.766 | -4.769 | -5.344 | -5.347 | -4.604 | -4.608 |
| **Cholecalciferol** | **D3** | -5.136 | -5.136 | -2.698 | -2.698 | -3.955 | -3.955 | -4.646 | -4.646 |
| **Cyanocobalamin** | **B12** | -9.05 | -9.117 | -11.15 | -11.218 | -10.362 | -10.43 | -8.525 | -8.592 |
| **Ergocalciferol** | **D2** | -3.703 | -3.703 | -4.847 | -4.847 | -4.027 | -4.027 | -4.892 | -4.892 |
| **Folic Acid** | **B9** | -7.26 | -8.379 | -6.781 | -7.06 | -6.488 | -6.768 | -4.079 | -4.359 |
| **Hydroxocobalamin** | **B12** | -9.05 | -9.117 | -11.15 | -11.218 | -10.365 | -10.432 | -8.525 | -8.592 |
| **Methylcobalamin** | **B12** | -7.488 | -8.811 | -10.641 | -10.711 | -8.811 | -8.881 | -8.218 | -8.288 |
| **Niacinamide** | **B3** | -5.467 | -5.467 | -5.908 | -5.908 | -4.79 | -4.79 | -5.45 | -5.45 |
| **Pantothenic Acid** | **B5** | -2.352 | -2.353 | -2.128 | -2.129 | -1.956 | -1.957 | -1.439 | -1.44 |
| **Phytomenadione** | **K1** | -4.213 | -4.213 | -3.508 | -3.508 | -2.96 | -2.96 | -3.414 | -3.414 |
| **Pyridoxal** | **B6** | -4.633 | -4.672 | -6.367 | -6.406 | -5.155 | -5.194 | -4.825 | -4.864 |
| **Retinol** | **A** | -2.962 | -2.962 | 0 | 0 | -3.084 | -3.084 | -4.32 | -4.32 |
| **Riboflavin** | **B2** | -5.673 | -5.673 | -5.456 | -5.456 | -5.706 | -5.706 | -5.11 | -5.111 |
| **Thiamine** | **B1** | -5.231 | -5.25 | -5.56 | -5.579 | -4.764 | -4.783 | -4.19 | -4.209 |
| **Tocopherols** | **E** | -4.086 | -4.086 | -2.739 | -2.739 | -3.578 | -3.578 | -4.241 | -4.241 |
| **Tocopheryl Acetate** | | -3.267 | -3.267 | -2.996 | -2.996 | -2.822 | -2.822 | -3.42 | -3.42 |
| **Vitamin K3** | **K3** | -4.715 | -4.715 | -5.933 | -5.933 | -3.869 | -3.869 | -4.838 | -4.838 |

Tables S4 : Binding energies (as Docking and Glide scores) of the tested vitamins against human cell receptors correlated with the three coronaviruses. All score values are in negative charge, as performed by Glide software (Schrödinger Release 2020-3: Glide; Schrödinger, LLC, New York, NY, USA, 2020). Protein 3D structures imported from protein database (PDB).

| | | ACE2 | | DPP4 | | hANP | |
|---|---|---|---|---|---|---|---|
| **chemical name** | **vitamine** | **docking score (kcal/mol)** | **Glide score (kcal/mol)** | **docking score (kcal/mol)** | **Glide score (kcal/mol)** | **docking score (kcal/mol)** | **Glide score (kcal/mol)** |
| **alfacalcidol** | **D3** | -5.779 | -5.779 | -4.371 | -4.371 | -4.028 | -4.028 |
| **alpha lipoic acid** | | -2.719 | -2.722 | -4.001 | -4.004 | -2.812 | -2.815 |
| **ascorbic acid** | **C** | -3.76 | -3.76 | -4.748 | -4.748 | -3.992 | -3.992 |
| **benfotiamine** | **B1** | -3.195 | -4.393 | -4.3 | -4.435 | -2.091 | -4.805 |
| **biotin** | **B7** | -4.888 | -4.888 | -5.097 | -5.1 | -4.049 | -4.053 |
| **cholecalciferol** | **D3** | -5.495 | -5.495 | -3.541 | -3.541 | -3.103 | -3.103 |
| **cyanocobalamin** | **B12** | -8.267 | -9.589 | -8.695 | -8.763 | -8.734 | -8.801 |
| **ergocalciferol** | **D2** | -5.552 | -5.552 | -3.851 | -3.851 | -3.764 | -3.764 |
| **folic acid** | **B9** | -4.497 | -4.776 | -5.964 | -7.083 | -5.222 | -5.502 |
| **hydroxocobalamin** | **B12** | -8.267 | -9.589 | -8.394 | -8.462 | -8.734 | -8.801 |
| **methylcobalamin** | **B12** | -6.817 | -6.887 | -7.416 | -7.486 | -9.222 | -9.291 |
| **niacinamide** | **B3** | -5.046 | -5.046 | -5.529 | -5.529 | -4.409 | -4.409 |
| **pantothenic acid** | **B5** | -2.571 | -2.572 | -2.194 | -2.95 | -0.624 | -0.625 |
| **phytomenadione** | **K1** | -3.504 | -3.504 | -2.895 | -2.895 | -3.372 | -3.372 |
| **pyridoxal** | **B6** | -4.59 | -4.629 | -5.73 | -5.769 | -3.671 | -3.709 |
| **retinol** | **A** | -5.404 | -5.404 | -3.191 | -3.191 | -3.179 | -3.179 |
| **riboflavin** | **B2** | -4.766 | -4.766 | -5.583 | -5.584 | -5.108 | -5.108 |
| **thiamine** | **B1** | -6.187 | -6.206 | -5.639 | -5.658 | -5.294 | -5.313 |
| **tocopherols** | **E** | -4.342 | -4.342 | -3.446 | -3.446 | -2.58 | -2.58 |
| **tocopheryl acetate** | | -3.44 | -3.44 | -3.903 | -3.903 | -3.17 | -3.17 |
| **vitamin k3** | **K3** | -3.946 | -3.946 | -4.89 | -4.89 | No interaction | No interaction |

Figure S1: antiviral activities of B-Com against SARS-CoV-2, and MERS-CoV by plaque reduction assay. Purchased B-Com ampule vials for injection are manufactured my Amoun Pharmaceutical Industries Company - Egypt. The table shows the composition of vitamins mix in the B-Com and the concentration of each in milli moles. It also shows the prepared dilutions (25,12.5,6.25, 3.125 micro liters per ml), and the concentration of each vitamin in the mix dilutions prepared in microliters.

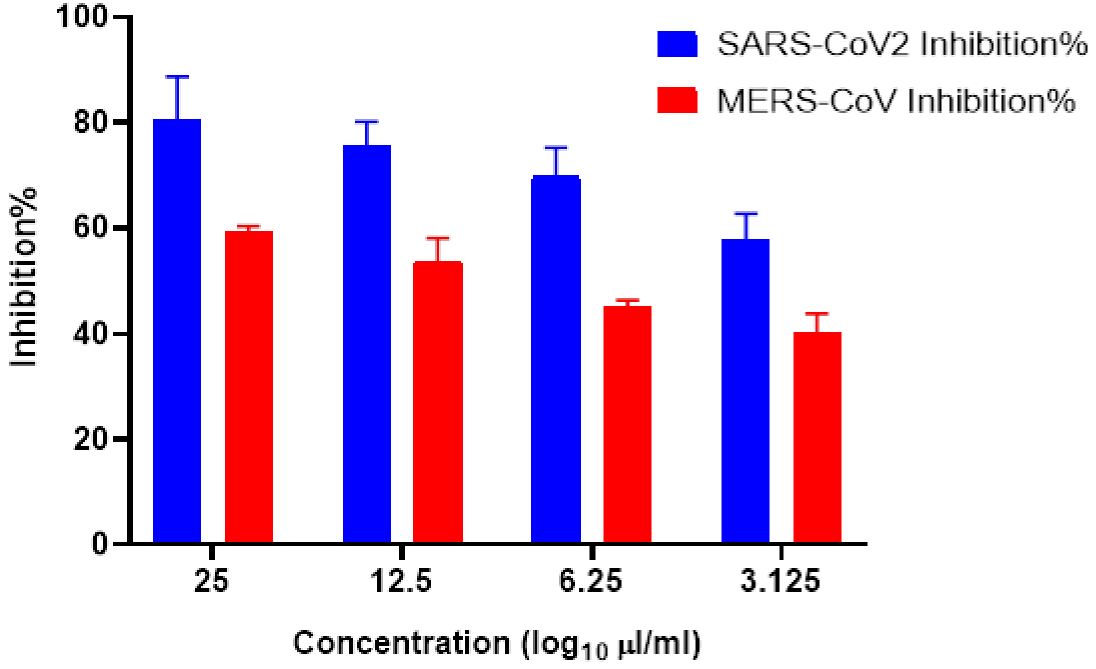


| B -Com Ampules By Amoun Pharmaceutical- Egypt | | | | | | |
|---|---|---|---|---|---|---|
| Vitamins in B-Com ampules * | | stock concentration / mmol | Tested Dilutions | | | |
| | | | 25 µl/ml | 12.5 µl/ml | 6.25µl/ml | 3.125µl/ml |
| | | | Concentrations /µmol | | | |
| Thiamine | B1 | 16.81 | 21.01 | 10.50 | 5.25 | 2.63 |
| Riboflavin | B2 | 5.71 | 7.14 | 3.57 | 1.79 | 0.89 |
| Nicotinamide | B3 | 163.77 | 204.71 | 102.36 | 51.18 | 25.59 |
| pyridoxine | B6 | 9.75 | 12.19 | 6.10 | 3.05 | 1.52 |
| D-Panthenol | B5 | 14.62 | 18.27 | 9.14 | 4.57 | 2.28 |

* Composition and Concentrations as provided by https://www.amoun.com/
